# Editorial: Molecular and cellular mechanisms for cancer therapy resistance

**DOI:** 10.3389/fonc.2023.1340318

**Published:** 2023-11-29

**Authors:** Anna Martina Battaglia, Emanuele Giorgio, Lavinia Petriaggi, Flavia Biamonte, Majid Momeny

**Affiliations:** ^1^ Department of Clinical and Experimental Medicine, Magna Græcia University of Catanzaro, Catanzaro, Italy; ^2^ The Brown Foundation Institute of Molecular Medicine, McGovern Medical School, The University of Texas Health Science Center at Houston, Houston, TX, United States

**Keywords:** cancer therapy resistance, ER stress, microRNAs, radiation therapy, chemoresistance

Despite advances in cancer therapeutic strategies, evolution of resistance is a major obstacle and limits the efficacy of different therapeutic approaches (1). Combination therapy yields a better anti-tumor activity and reduces the likelihood for tumor recurrence, however, innate and acquired resistance to combination strategies occur, especially in patients with metastatic disease (2). Identification of certain mechanisms of resistance has helped development of alternative therapies with a better clinical benefit, indicating that an improved understanding of the mechanisms driving resistance is of paramount importance (2). The current Research Topic aimed to provide the most recent findings about resistance mediators and novel approaches to tackle therapy resistance (**Figure 1**).

**Figure 1:**
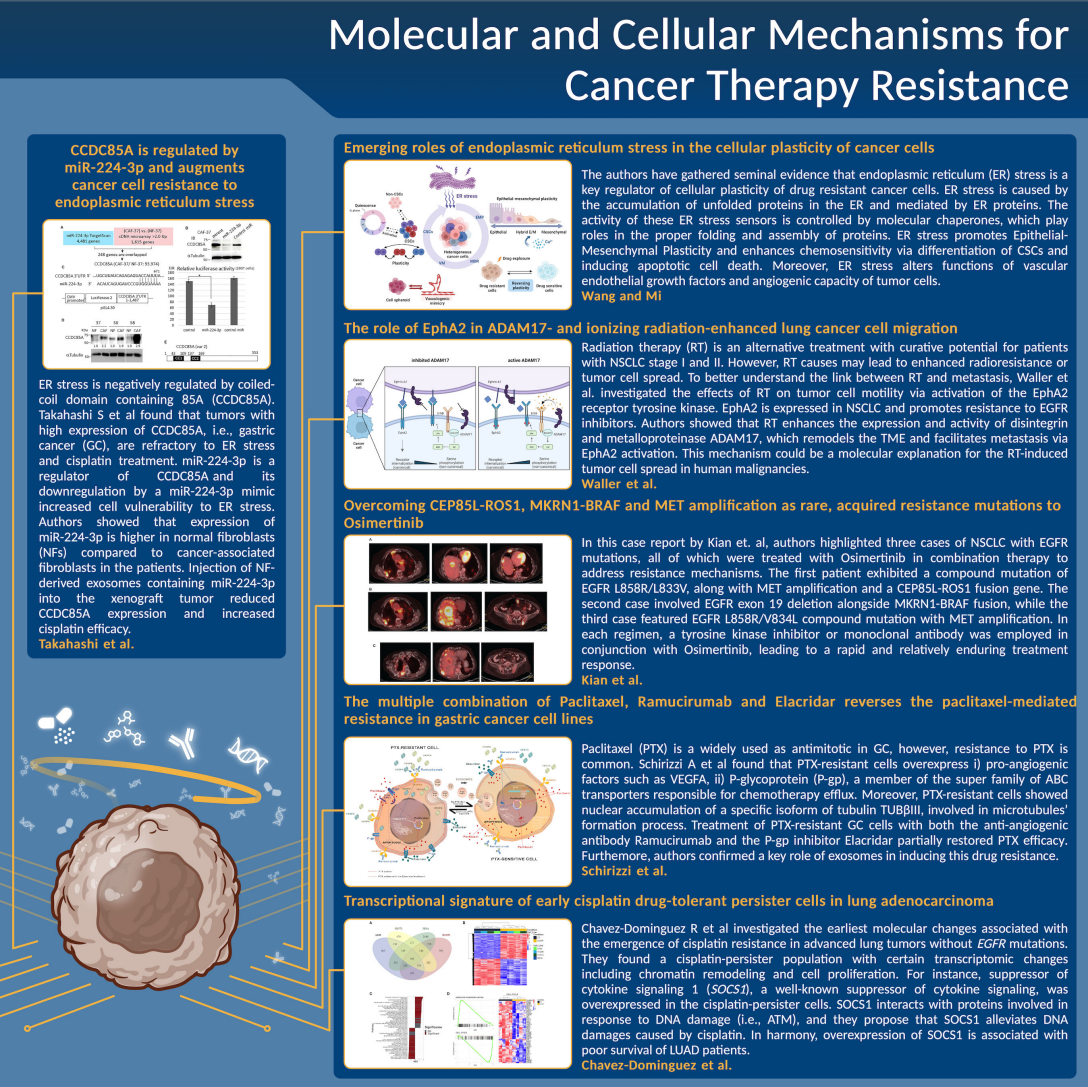
A schematic summary of the main results, issues, and conclusions about the novel approaches to tackle cancer therapy resistance for each manuscript discussed in this Editorial.

Wang and Mi have gathered evidence that endoplasmic reticulum (ER) stress is a key regulator of cellular plasticity and thereby, therapy resistance. ER stress is mediated by ER proteins, i.e., activating transcription factor 6 (ATF6), which assists cell survival (3). ER stress promotes epithelial-mesenchymal plasticity (EMP) via activation of EMP-inducing signaling pathways (4). On the other hand, ER stress enhances chemosensitivity via differentiation of cancer stem cells (5) and inducing apoptosis (6). Moreover, ER stress alters functions of vascular endothelial growth factors and angiogenic capacity of tumor cells (7).

ER stress is negatively regulated by coiled-coil domain containing 85A (CCDC85A). Takahashi et al found that tumors with high expression of CCDC85A, i.e., gastric cancer (GC), are refractory to ER stress and cisplatin treatment. miR-224-3p is a regulator of CCDC85A and downregulation of CCDC85A by a miR-224-3p mimic increased cell vulnerability to ER stress. The authors showed that expression of miR-224-3p is higher in normal fibroblasts (NFs) compared to cancer-associated fibroblasts in the patients. Injection of NF-derived exosomes containing miR-224-3p into the xenograft tumor reduced CCDC85A expression and increased cisplatin efficacy, suggesting that miR-224 and CCDC85A are promising targets to prevent cisplatin resistance.

Paclitaxel (PTX) is a widely used as antimitotic in GC, however, resistance to PTX is common (8). By performing differential expression analysis between PTX-resistant GC cell lines and sensitive ones, Schirizzi et al found that PTX-resistant cells overexpress i) pro-angiogenic factors such as VEGFA, ii) P-glycoprotein (P-gp), a member of the super family of ABC transporters responsible for chemotherapy efflux. Moreover, PTX-resistant cells showed nuclear accumulation of a specific isoform of tubulin TUBβIII, involved in microtubules’ formation process (9, 10). Treatment of PTX-resistant GC cells with both the anti-angiogenic antibody Ramucirumab and the P-gp inhibitor Elacridar partially restored PTX efficacy (Schirizzi et al). The majority of the resistance mediators are spread in the TME via exosomes (11). It was shown in this study that exosomes from PTX-resistant GC cells overexpress VEGFA and P-gp compared to those from sensitive GC cells. Moreover, PTX-sensitive cells acquire characteristics of the resistant cells when treated with the supernatant of the PTX-resistant GC cells, thus confirming key roles of exosomes in inducing drug resistance (Schirizzi et al).

Lung cancer is the leading cause of cancer-related death with a 5-year survival rate of 10-14% (12). Inhibitors of epidermal growth factor receptor (EGFR) tyrosine kinase such as Osimertinib are first-line treatments for non-small cell lung cancer (NSCLC) with *EGFR* mutations (13). Despite promising initial responses, almost all patients develop resistance via new *EGFR* mutations or activation of compensatory signaling pathways (14). In a case report by Kian et al, the authors have reported 3 different cases who were treated with osimertinib in a combination therapy to overcome resistance. The first case had a L858R/L833V mutation, MET amplification, and a CEP85L-ROS1 fusion gene with multiple liver metastases. After disease progression on osimertinib, the MET inhibitor crizotinib was added to the treatment and the combination therapy decreased the liver mass. The second case exhibited an exon 19del and an MKRN1-BRAF fusion. Following disease progression, the BRAF kinase inhibitor trametinib and dabrafenib were added to osimertinib, which yielded a partial response. The last case showed an *EGFR* L858R/V834L mutation with MET amplification. Upon disease progression on osimertinib, the bispecific EGFR and MET-directed antibody amivantamab was added to osimertinib, which exhibited a partial response. Unfortunately, all the patients passed away due to disease progression, implying for alternative mechanisms for therapy resistance.

Cisplatin is the standard care treatment in advanced lung tumors without *EGFR* mutations, although development of resistance is inevitable (15). Chavez-Dominguez et al investigated the earliest molecular changes associated with the emergence of cisplatin resistance. To do this, they performed RNA sequencing of lung adenocarcinoma cell lines after cisplatin treatment and found a cisplatin-persister population with certain transcriptomic changes including chromatin remodeling and cell proliferation. For instance, suppressor of cytokine signaling 1 (*SOCS1*), a well-known suppressor of cytokine signaling, was overexpressed in the cisplatin-persister cells. SOCS1 interacts with proteins involved in response to DNA damage (i.e., ATM) (16), and they propose that SOCS1 alleviates DNA damages caused by cisplatin. In harmony, overexpression of SOCS1 is associated with poor survival of LUAD patients. This study highlights SOCS1 as a response biomarker of cisplatin treatment and as a potential target to overcome drug resistance.

Radiation therapy (RT) is an alternative treatment with curative potential for patients with NSCLC stage I and II who are inoperable or refuse surgery (17). RT causes DNA double-strand breaks and ultimately cell death, however, it may modulate the TME and leads to enhanced radioresistance or tumor cell spread (18). To better understand the link between RT and metastasis, Waller et al. investigated the effects of RT on tumor cell motility via activation of the EphA2 receptor tyrosine kinase Fare clic o toccare qui per immettere il testo.. EphA2 is expressed in NSCLC and promotes resistance to EGFR inhibitors (19). In their mechanistic *in vitro* study, the authors showed that RT enhances the expression and activity of disintegrin and metalloproteinase ADAM17, which remodels the TME and facilitates metastasis via EphA2 activation. This mechanism could be a molecular explanation for the RT-induced tumor cell spread in human malignancies.

Taken together, the interesting studies published in this Research Topic point out that there is a pressing need to further apprehend the dynamics of tumor adaptation in response to treatment in order to significantly improve the current therapies.

## Author contributions

AMB: Writing – original draft. EG: Writing – original draft. LP: Writing – original draft. FB: Writing – original draft. MM: Writing – original draft.
